# Simple Isothermal and Label-Free Strategy for Colorectal Cancer Potential Biomarker miR-625-5p Detection

**DOI:** 10.3390/bios13010078

**Published:** 2023-01-02

**Authors:** Yifei Chen, Lizhen Ye, Hui Chen, Tingting Fan, Cheng Qiu, Yan Chen, Yuyang Jiang

**Affiliations:** 1 State Key Laboratory of Chemical Oncogenomics, Guangdong Provincial Key Laboratory of Chemical Biology, Tsinghua Shenzhen International Graduate School, Shenzhen 518055, China; 2 School of Pharmaceutical Sciences, Health Science Center, Shenzhen University, Shenzhen 518060, China; 3 School of Pharmaceutical Sciences, Tsinghua University, Beijing 100084, China

**Keywords:** colorectal cancer, miR-625-5p, strand displacement amplification, SYBR Gold

## Abstract

miRNA is considered a novel biomarker for cancer diagnosis and due to its low level in vivo, the development of new detection methods for it has become a research hotspot in recent years. Here, we firstly found that miR-625-5p was significantly upregulated in colorectal cancer tissues by means of differential expression analysis of the dbDEMC database and clinical validation. Subsequently, it was found that miR-625-5p promoted cell proliferation and migration but inhibited apoptosis through phenotypic experiments; thus, we initially identified miR-625-5p as a potential biomarker for colorectal cancer. Moreover, in order to monitor slight changes in the miR-625-5p level, we developed a novel detection method for it based on strand displacement amplification (SDA). In this system, a hairpin was designed to recognize and pair with miR-625-5p, which was used as a primer to initiate SDA, and a large number of complementary DNAs were generated via cyclic amplification, followed by the addition of SYBR Gold to achieve quantitative analysis of miR-625-5p. Moreover, this method showed a good response to miR-625-5p with a detection limit of 8.6 pM and a dynamic range of 0.01 to 200 nM, and the specificity of it was verified using a set of other miRNAs as an interference. Finally, we set up different concentrations of biologic samples for detection to verify the practicability of the method. The results of this study indicate that this detection method has great potential in clinical diagnosis.

## 1. Introduction

Colorectal cancer (CRC) is currently the third most common cancer in the world and has the second highest mortality rate [1]. Early diagnosis of colorectal cancer is very important to reduce mortality, but more than 60% of patients are difficult to diagnose when the tumor is still localized [2,3]. At present, the traditional detection methods, such as fecal occult blood tests and endoscopy, cause great harm to the patient’s body [4], and the results might show high false positive and false negative rates [5,6]. The discovery of biomarkers has made it possible to carry out a more personalized treatment plan according to the specific gene expression of patients, thereby improving the specificity of diagnosis [7]. Most of the selected biomarkers are derived from tissues and serum, which is less invasive, or even noninvasive, to patients [8]. Therefore, finding new biomarkers and developing corresponding detection methods are currently the hotspots of cancer research.

Micro-RNA (miRNA) is the most studied class of noncoding RNA, which is involved in the negative regulation of more than 60% of protein-coding gene expression [9]. In recent years, more and more studies have shown that miRNA plays an important regulatory role in the occurrence and development process of colorectal cancer and metastasis to liver cancer [10,11], and considering its high stability in many biological samples, miRNA is also regarded as a new candidate of tumor biomarkers [12,13].

The expression level of miRNA in vivo is relatively low [14]. Although typical real-time quantitative PCR (RT-qPCR) can also quantitatively analyze it, the detection results may be false positive for specific miRNA levels, and the process is time-consuming and depends on specific instruments [15,16]. In order to improve the effect of detecting specific miRNA, more and more new detection principles have been proposed, such as rolling circle amplification (RCA) [17,18], strand displacement amplification (SDA) [19,20] and catalyzed hairpin assembly amplification (CHA) [21]. Although these methods can improve efficiency and do not depend on specific instruments, RCA might generate non-specific amplification [22] and CHA is limited by an uncatalyzed reaction called circuit ‘leakage’ [23]. In comparison, SDA has good efficiency in signal amplification and good sensitivity even at the single-cell level, and it can achieve real-time assays in some cases [24]. The SDA reaction generally requires polymerase and endonuclease, which takes advantage of the extension and nicking of specific sites to generate a large number of detection fragments [25], and then the released fragments can be detected by SYBR Gold. Quantitative analysis of target miRNA can be performed according to the fluorescence signal. Previous studies have shown that SDA with the assistance of SYBR Gold could achieve effective detection of ATP [26].

In this study, we first used the results of differential expression analysis of miRNA expression profiles in the dbDEMC database [27] to find that miR-625-5p was significantly upregulated in colorectal cancer and then extracted miRNAs of the tumor and adjacent tissues (5 cm away from the tumor) from 24 CRC patients for clinical validation. Next, we performed a series of phenotypic experiments to verify its oncogenic function in CRC. Moreover, a new detection method was developed for miR-625-5p based on SDA. We designed a hairpin, whose 3’ terminal sequence contains a part that could complement and pair with miR-625-5p. Then, miR-625-5p was used as a primer to be extended and nicked at a specific site under the action of enzymes, and a large number of detection fragments were generated via cyclic amplification. Finally, SYBR Gold was added to detect the miRNA level. Compared with the traditional method, our reaction is basically carried out at 37 °C, which is simple and rapid, and our system showed a good response to miR-625-5p with a detection limit of 8.6 pM and a dynamic range of 0.01 to 200 nM; its specificity was verified using a set of other miRNAs as an interference. In order to verify the practicability of the method, we set up different concentrations of samples for detection. Our results show that this detection method has great potential in clinical diagnosis.

## 2. Materials and Methods

### 2.1. Cell Transfection

miR-625-5p mimics, inhibitor, mimics-NC and inhibitor-NC were synthesized by Genepharma Company (Suzhou, China). The transfection reagent was Invi DNA/RNA transfection reagent developed by Invigentech Company (Irvine, CA, USA). According to the recommended dosage of the manufacturer’s instructions, it was fully mixed with the RNA to be transfected at a ratio of 1:1 and added after 15 min, and then the culture was changed after 24 h.

### 2.2. CCK-8 Assay

After 24 h of the above transfection, the cells were digested with trypsin (Beyotime, Shanghai, China), forming cell suspension. The cells were seeded into a 96-well plate (Costar, Corning, NY, USA), with about 3000 cells per well. An amount of 10 μL CCK-8 (Yeasen, Shanghai, China) was added to each well after 24 h, 48 h and 72 h of incubation, and then the OD450 of each well was detected using a microplate reader (Tecan infinite M1000 Pro, Männedorf, Switzerland) after 2 h of incubation. Additionally, triplicate wells with relatively close values were selected for subsequent data analysis.

### 2.3. Colony Formation Assay

After the transfection was completed, the cells were digested with trypsin, forming cell suspension. The cells were seeded into a 6-well plate (Costar, Corning, NY, USA), with about 500 cells in each well. After 2 weeks of incubation, the culture was removed, rinsed with 1× PBS (Beyotime, Shanghai, China), fixed with methanol (Xilong Scientific Co., Ltd., Shantou, China) for 40 min, stained with crystal violet (Beyotime, Shanghai, China) for 15 min, slowly washed with water, and dried. Finally, it was photographed by a smartphone.

### 2.4. Transwell Assay

Approximately 10^6^ cells were collected 24 h after transfection, resuspended in 300 μL serum-free medium and added to the 8 μm upper chamber, followed by the addition of 700 μL medium containing 20% FBS (Gibco, Grand Island, NY, USA) to the lower chamber. After 36–48 h, cells that migrated to the lower chamber were fixed with methanol (Xilong Scientific Co., Ltd., Shantou, China) for 40 min, stained with crystal violet (Beyotime, Shanghai, China) for 15 min, rinsed in 1× PBS, dried and photographed under an inverted microscope.

### 2.5. Apoptosis Assay

A suspension of cells (1 × 10^5^ cells/well) in the logarithmic growth phase was seeded in a 6 cm cell culture plate (Costar, Corning, NY, USA) and incubated for 12 h in a 37 °C incubator containing 5% CO_2_. After transfection, the cells were cultured for 48 h. After digestion with trypsin without EDTA and washing with 1× PBS, the cells were resuspended in 100 μL of 1× Binding Buffer. Then, 5 μL Annexin V-FITC and 10 μL PI Staining Solution (Yeasen, Shanghai, China) were added, and the reaction was kept in the dark at room temperature for 15 min. Then, 400 μL of 1× Binding Buffer was added, mixed and placed on ice. Samples were detected via flow cytometry within 1 h.

### 2.6. RT-qPCR

Tissues were collected from 24 CRC patients in Shenzhen Second People’s Hospital. miRNAs were extracted from tissues using RNAiso for Small RNA Reagent from Takara Biomedical Technology (Beijing) Co., Ltd. (Beijing, China) according to the corresponding instructions. The extracted miRNAs were reversed into cDNAs using the Mir-X miRNA First-Strand Synthesis Kit (Takara Biomedical Technology (Beijing) Co., Ltd., Beijing, China). Next, the qPCR reaction system was configured according to the product instructions of 2× SYBR Green qPCR Master Mix (Bimake, Houston, TX, USA). Then, the Applied Biosystems 7500 Real-Time PCR System (Thermofisher, Waltham, MA, USA) was used to detect the relative expression level of miR-625-5p in the above qPCR reaction system, and U6 snRNA was used as the internal reference. The relative expression level of miR-625-5p was calculated using the $2^{{-(\mathrm{Ct}}_{\mathrm{miR}-625-5p}{-\mathrm{Ct}}_{U6})}$ method.

### 2.7. Western Blot Analysis

After 72 h of transfection, the proteins were extracted with RIPA lysate (Beyotime, Shanghai, China) and then quantified using a BCA Protein Assay kit (Beyotime, Shanghai, China). Modified 5× loading buffer was added to the samples, and the samples were denatured at 95 °C for 10 min before loading. After washing with 1× TBST, the primary antibody was incubated at 4 °C overnight. The primary antibody was recycled and then the PVDF membrane was washed with 1× TBST. After incubating with the secondary antibody for 2 h at room temperature, the ECL method was used to develop and photograph the membrane.

### 2.8. miRNA Detection Reagents

Deoxy-ribonucleoside triphosphates (dNTPs) were bought from Sangon Biotechnology Co., Ltd. (Shanghai, China). Klenow Fragment polymerase (3′-5′exo-, KF polymerase) and the nicking endonuclease Nt.BbvCI were purchased from New England Biolabs Ltd. (Ipswich, UK). Tris-HCl, NaCl and MgCl_2_ were obtained from Beyotime Co., Ltd. (Shanghai, China), DNA sequences were synthesized by Sangon Biotechnology Co., Ltd. (Shanghai, China). The sequence list is shown in Table 1. The stock solution of the hairpin (HP) was prepared by dissolving the lyophilized powder in 20 mM Tris-HCl buffer (100 mM NaCl, 5 mM MgCl_2_, pH 7.4). The HP was heated to 95 °C for 5 min and then cooled down to room temperature to form a suitable hairpin structure. The isothermal reaction buffer contained NEBuffer 2 and rCutsmart buffer (1:1).

### 2.9. miRNA Detection Apparatus

All fluorescence measurements were carried out in a 96-well assay plate (Costar, Coring, NY, USA) using a microplate reader (Tecan infinite M1000 Pro, Männedorf, Switzerland) at room temperature. The excitation wavelength was set to 480 nm, and the emission spectra from 500 to 700 nm were collected. The fluorescence intensity at 545 nm (for SYBR Gold) was used to evaluate the performance of the designed method.

### 2.10. Optimization of the Reaction Conditions

All reaction conditions were optimized including the concentrations of dNTPs, KF polymerase and Nt.BbvCI, and the reaction time of 2 steps (Step 1: the HP combined with miR-625-5p; Step 2: isothermal amplification). The concentration range of dNTPs was 125–500 nM. The selected KF polymerase amount was 2.5–6.25 U. The Nt.BbvCI amount was 5–20 U. The reaction time range was 2–4 h. While optimizing the reaction conditions, only one condition was changed at once, and the other conditions remained unchanged.

### 2.11. miR-625-5p Assay

The experiments were carried out in the 2 steps described above. Step 1 proceeded with 20 μL of solution containing 2 μL of the HP (final reaction concentration, 40 nM), 13 μL Tris-HCl buffer and 5 μL of target miRNA (various concentrations ranging from 0 to 300 nM), followed by incubation at 37 °C for 1 h. Subsequently, Step 2 was carried out with 50 μL of solution containing 20 μL of the solution from Step 1, 5 μL NEBuffer 2, 5 μL rCutsmart buffer, 5 μL dNTPs (250 nM), 0.5 μL KF, 1 μL Nt.BbvCI and 13.5 μL DEPC water, followed by incubating at 37 °C for 1 h. Then, the reaction was terminated via incubation at 80 °C for 20 min. Next, 1× SYBR Gold was added into the products of the above reaction. After incubation at room temperature for 5 min, the samples were used for fluorescence measurements. In order to verify the selectivity of the proposed strategy, five interfering miRNAs including miR-203b-5p, miR-219a-2-3p, miR-130a-3p, miR-4661-5p and miR-561-5p used for comparison with miR-625-5p. The experimental conditions used were in accordance with the optimized result above. The concentrations of miR-625-5p and other interfering miRNAs were 100 nM.

### 2.12. Assay of miR-625-5p for Biologic Samples

miRNAs were extracted from HT-29 cells using RNAiso for Small RNA Reagent from Takara Biomedical Technology (Beijing) Co., Ltd. (Beijing, China) according to the corresponding instructions. Then, the extracted miRNAs were diluted with DEPC water (Beyotime, Shanghai, China) from 0 to 80 ng/μL and the fluorescence intensity of each sample was detected after the reaction as described above.

## 3. Results

### 3.1. The Discovery of a Novel Potential CRC Biomarker miR-625-5p

**Figure 1:**
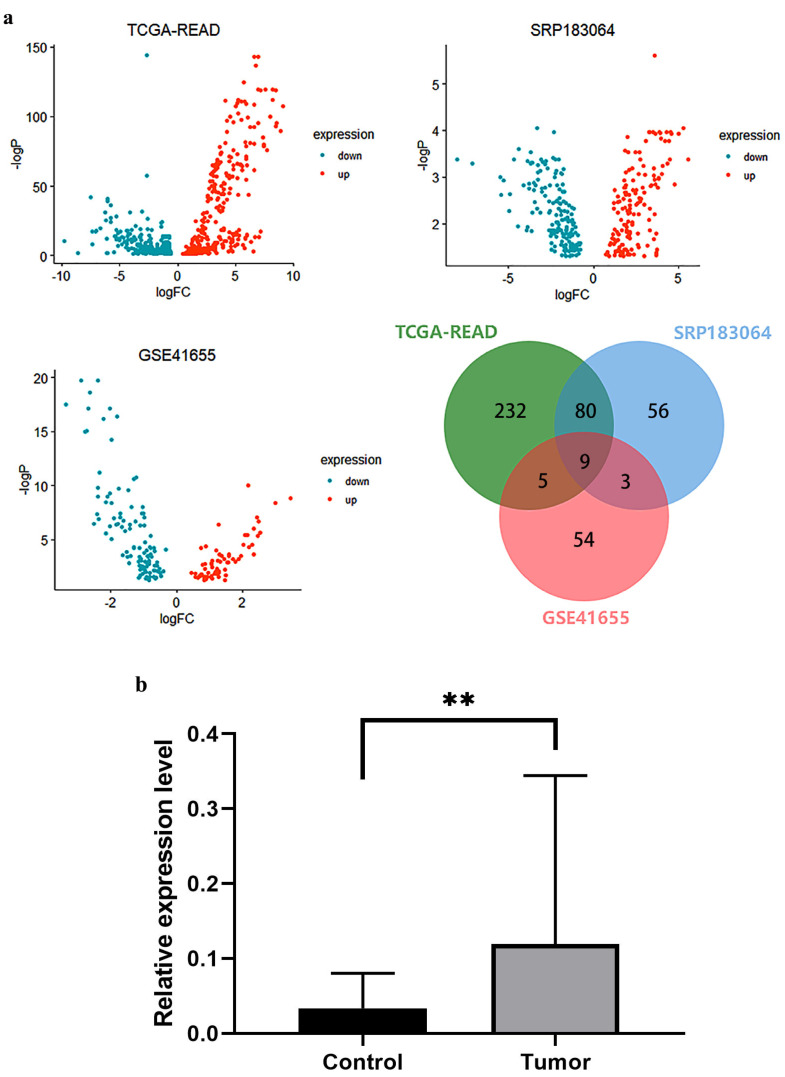
Bioinformatic analysis and clinical validation for preliminary screening of CRC biomarker. (**a**) Three datasets (TCGA−READ, SRP183064 and GSE41655) chosen for differential expression analysis and drawn by volcano plot (up- and downregulated miRNAs) and Venn diagram (upregulated miRNAs). (**b**) The relative expression level of miR-625-5p between tumor and adjacent tissues (5 cm away from the tumor). Tumor represents the tumor tissues and Control represents the adjacent tissues. All data are presented as mean ± SD, n = 24, ** *p* < 0.01.

With the development of bioinformatics, more and more novel biomarkers of CRC have been discovered by making use of clinical sample data from multiple datasets in the TCGA and GEO databases. In this study, firstly, the TCGA-READ, GSE41655 and SRP183064 datasets were chosen for differential expression analysis using the dbDEMC database, and then we selected the up-regulated miRNAs from the three datasets, creating a Venn diagram using JVenn [28]. The intersections of genes with significant differential expression were considered to be candidate genes including hsa-miR-96-5p, hsa-miR-135b-5p, hsa-miR-21-5p, hsa-miR-625-5p, hsa-miR-148a-3p, hsa-miR-203a-3p, hsa-miR-224-5p, hsa-miR-200b-3p and hsa-miR-183-5p (Figure 1a). It was found that several candidate genes such as hsa-miR-21-5p and hsa-miR-135b-5p have been confirmed as CRC biomarkers [13] and used as targets for the development of subsequent detection methods. Furthermore, the tumor tissues and adjacent tissues (5 cm away from the tumor) of 24 CRC patients were selected to extract miRNA for detection. The results of RT-qPCR showed that the relative expression level of miR-625-5p was significantly upregulated in CRC tumor tissues (Figure 1b). At present, miR-625-5p has been identified as a novel biomarker of immunotherapy response in advanced non-small-cell lung cancer patients [29], and miR-625-3p, which is another product of miR-625 modification, promotes cell migration and invasion via inhibition of SCAI in colorectal carcinoma cells [30]; however, there are few studies on miR-625-5p in colorectal cancer, and its biological function is not clear. Based on the results of bioinformatic analysis and clinical validation, it is preliminarily believed that the expression of miR-625-5p is significantly upregulated in CRC and may play a role in promoting cancer progression.

In order to explore the biological functions of miR-625-5p, the mimics and inhibitor of miR-625-5p and the corresponding negative control sequences (mimics-NC and inhibitor-NC) were designed and synthesized, and then transfected into the cells.

**Figure 2:**
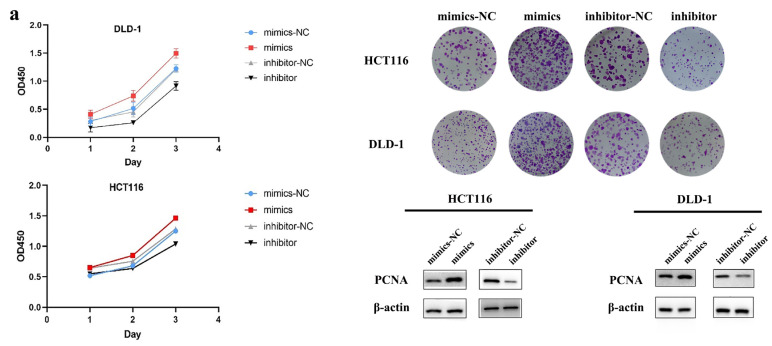

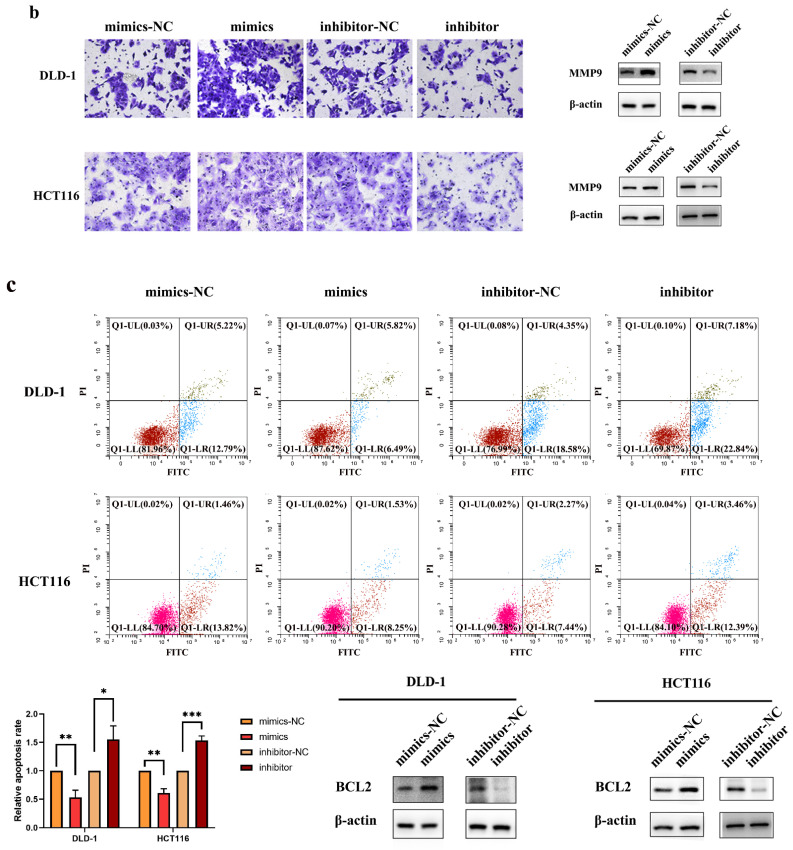
The assays of cell proliferation, transwell and apoptosis on DLD-1 and HCT116. (**a**) The cell proliferation curves, colony formation assays and PCNA expression level, the values of OD450 are expressed as mean ± SD, the pictures of colony formation were taken by a smartphone, the diameter of the well is about 34.8 mm; (**b**) the transwell assays and MMP9 expression level, the pictures of transwell were taken by a 20× objective and a 10× eyepiece; (**c**) the cell apoptosis assays and BCL2 expression level. All assays contained mimics, mimics-NC, inhibitor, inhibitor-NC group. The relative apoptosis rate is expressed as mean ± SD, * *p* < 0.05, ** *p* < 0.01, *** *p* < 0.001. Each assay was performed in triplicate.

For cell proliferation, after 24 h, 48 h and 72 h of incubation, CCK-8 was added to detect cell viability by measuring the OD450, and the proliferation curves were drawn. The results showed that after 24 h, the cell proliferation rate of each group was similar, but from 48 h to 72 h, the cell proliferation rate of the mimics group was significantly higher than that of the mimics-NC group (Figure 2a). For the inhibitor group, the cell proliferation rate was significantly decreased after miR-625-5p was downregulated (Figure 2a). In addition, in order to confirm its effect on cell proliferation, we also performed colony formation assays. The results showed that the amount of colony formation after transfection with mimics was increased compared with the control; however, for the inhibitor, it was lower than that of the control, indicating that miR-625-5p can promote cell proliferation and accelerate colony formation (Figure 2a). In addition, we also performed Western blotting to compare the expression level of PCNA. As shown in the figure below, the expression level of the PCNA protein was upregulated after transfecting the mimics, while after transfecting inhibitor, the expression level of PCNA protein decreased (Figure 2a). Therefore, miR-625-5p may promote CRC cell proliferation.

Moreover, it was found that when miR-625-5p was overexpressed, the number of cells penetrating the upper chamber and entering the lower chamber was significantly higher than that of the control within 48 h, and the inhibitor group showed the opposite result (Figure 2b). In addition, the expression level of MMP9 in the mimics group also increased compared with that in the control group, while that of the inhibitor group decreased (Figure 2b). In conclusion, miR-625-5p may play an important regulatory role in promoting CRC cell migration, which also indicates that this miRNA may be related to tumor metastasis.

To investigate the effect of miR-625-5p on the apoptosis of CRC cells, we transiently transfected the mimics, inhibitor and corresponding negative controls. The expression level of BCL2, a marker protein that inhibits apoptosis, was upregulated in the mimics group but decreased with the downregulation of miR-625-5p. On the other hand, the number of apoptotic cells significantly decreased in the mimics group, while the number of apoptotic cells increased in the inhibitor group (Figure 2c), which means that miR-625-5p may play an inhibitory role in cell apoptosis. Therefore, miR-625-5p can promote the expression of BCL2 and inhibit the apoptosis process of tumor cells to promote the occurrence and development of CRC (Figure 2c).

### 3.2. The Principle of miR-625-5p Detection

**Figure 3:**
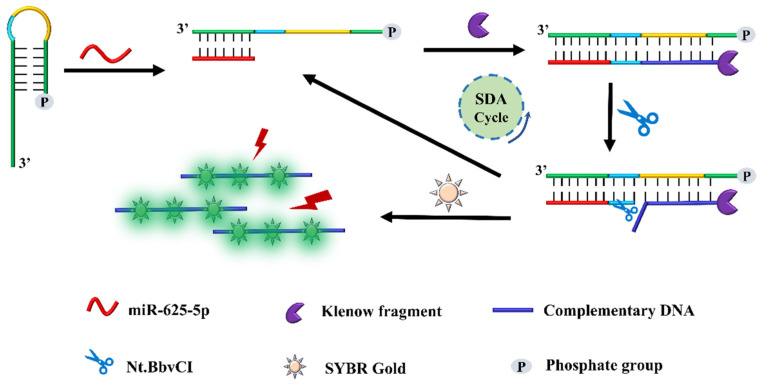
The principle of our detection method based on strand displacement amplification and SYBR Gold. miR-625-5p acts as a primer to open the stem loop of the hairpin and generate many complementary DNAs followed by the addition of KF polymerase, Nt.BbvCI enzyme and dNTPs. The complementary DNAs can be detected by SYBR Gold. The sequences of the hairpin and miR-625-5p can be seen in Table 1.

miR-625-5p may be considered as a potential tumor biomarker based on the above results. However, due to its relatively low content in cells, in order to detect it more efficiently, we developed a novel detection method, and its principle is shown in the following Figure 3. First, we designed a hairpin that contains two important components: a complementary fragment at the 3′ terminal that can specifically bind to miR-625-5p, and a specific sequence whose complimentary sequence can be recognized and cleaved by the nicking enzyme Nt.BbvCI. If the sample contains miR-625-5p, since the stem-loop sequence is complementary to the target sequence and the number of base-pairs is higher than the stem-loop itself, the stem-loop opens and forms complementary pairs. Then, miR-625-5p acts as a primer to be extended from its 5′ terminal and generated a double-stranded structure after adding KF polymerase and dNTPs. Subsequently, the Nt.BbvCI enzyme recognizes the specific site and produces a gap in the complementary DNA. Then, the complementary DNA is released, and the remaining part of the fragment re-enters the amplification cycle. Finally, the nucleic acid dye SYBR Gold is added for detection. In addition, it should be noted that the 5′ terminal of the hairpin needs to be phosphorylated, otherwise the hairpin structure can be extended from the 5’ to 3’ terminal under the catalysis of KF polymerase even if it is not opened, yielding false positive results. Different from previous methods, we used a hairpin instead of straight-strand cDNA to combine with the target at first, which strengthened the selectivity of the detection method. Additionally, we divided the binding reaction of the hairpin and miRNA and the subsequent amplification reaction into two steps, so that the hairpin can be fully opened and combined with miR-625-5p, which contributed to the following amplification reaction.

### 3.3. Verifying the Feasibility of the miR-625-5p Assay

**Figure 4:**
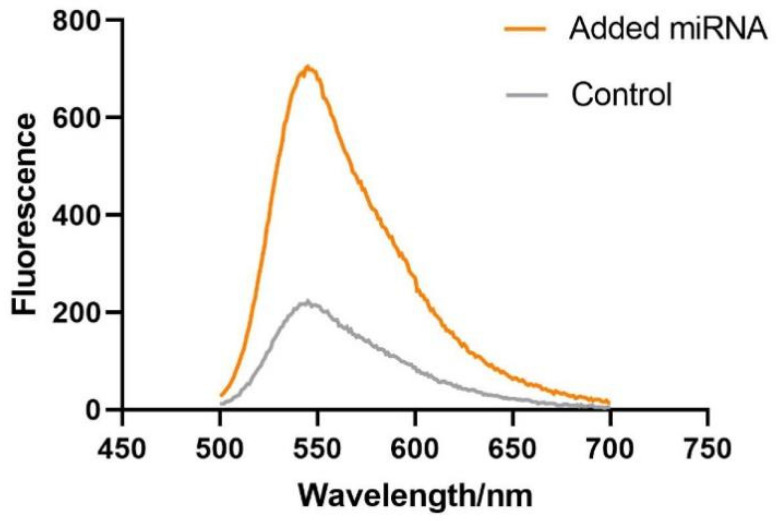
The feasibility assay of the detection method. Added miRNA: HP + miR-625-5p + KF polymerase + Nt.BbvCI + SYBR Gold; Control: HP + KF polymerase + Nt.BbvCI + SYBR Gold. The concentration of reagents above: HP (40 nM), miR-625-5p (100 nM), KF polymerase (5 U), Nt.BbvCI (20 U), SYBR Gold (1×).

In order to verify the feasibility of the method designed above, we measured the fluorescence emission spectra of the miR-625-5p of different groups. The result showed that the control, which did not contain any miRNA, merely had a low signal because the isothermal amplification was not initiated; however, after adding the target miR-625-5p, the stem-loop of the hairpin was opened and combined with the target, and initiated the isothermal strand displacement amplification, causing the fluorescence intensity to significantly increase (Figure 4). Therefore, this indicates that the proposed detection method is efficient for the detection of target miRNA.

### 3.4. Optimization of Reaction Conditions

**Figure 5:**
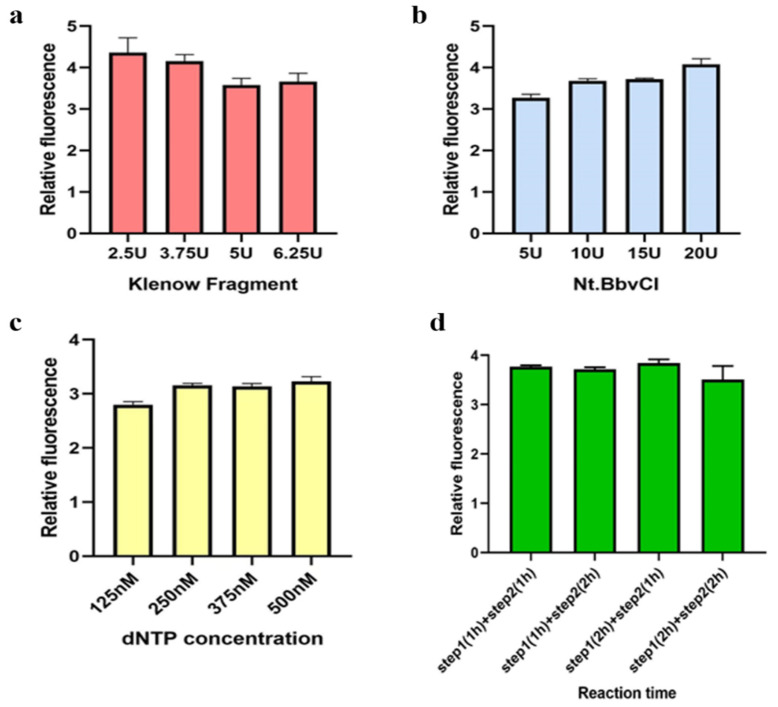
Optimization of the reaction conditions for the detection method of miR-625-5p. (**a**) Optimization of the amount of KF polymerase; (**b**) optimization of the amount of Nt.BbvCI; (**c**) optimization of the concentration of dNTPs; (**d**) optimization of the reaction time. The concentration of the other reagents remained unchanged when each reaction condition was optimized. All data are presented as mean ± SD. Each assay was performed in triplicate.

In this study, we mainly used two enzymes, KF polymerase and Nt.BbvCI. The amount of enzyme has an impact on the reaction effect. Additionally, considering the cost of the experiments, we first optimized the amount of enzymes to achieve the expected detection effect with less enzymes. We chose a range of 2.5–6.25 U to optimize KF polymerase, and as shown in Figure 5a,b; when 20 U of Nt.BbvCI enzyme and 2.5 U of KF polymerase were used, the best amplification effect could be observed. Then, we controlled the amount of KF polymerase to 2.5 U, and it was found that 10 U Nt.BbvCI enzyme could achieve a good detection effect. Therefore, 2.5 U of KF polymerase and 10 U of Nt.BbvCI enzyme were determined to optimize the concentration of dNTPs under the premise of the comprehensive consideration of the cost and experimental effect. Next, the optimized experiment of the reaction concentration of dNTPs was carried out according to the concentration range set in the methods section. When the final concentration of dNTPs reached 250 nM, the detection effect was similar to that with 500 nM (Figure 5c), so we selected 250 nM dNTPs to optimize the reaction time. Additionally, the result showed a reaction time of only 1 h was required for both the first step and second step to achieve a relatively good reaction effect (Figure 5d).

### 3.5. The Determination of the Detection Range and Limit

**Figure 6:**
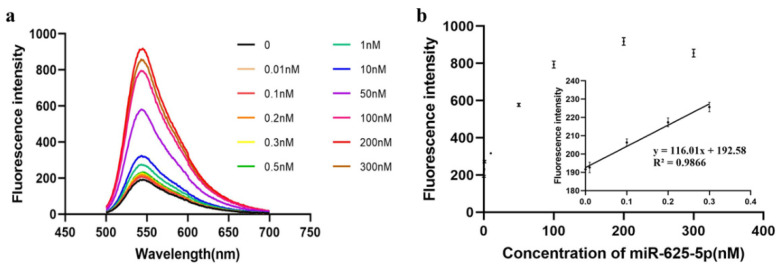
Determination of the detection range and limit. (**a**) The fluorescence emission spectra of samples with different concentrations of miR-625-5p ranging from 0 to 300 nM; (**b**) the plot of correlation between the concentration of miR-625-5p and the fluorescence intensity. The inset showed the concentration ranging from 0.01 to 0.3 nM had a linear relationship with the fluorescence intensity. The fluorescence intensities of different concentrations are presented as mean ± SD. Each assay was performed in triplicate.

The detection range and detection limit of miR-625-5p were determined according to the optimized reaction conditions. Firstly, the detection concentration gradient was set to 0, 0.01, 0.1, 0.2, 0.3, 0.5, 1, 10, 50, 100, 200 and 300 nM, and the fluorescence intensities of samples with different concentrations were detected (Figure 6a). The result showed that when the concentration of miR-625-5p reached 200 nM, its fluorescence intensity reached saturation. Next, we selected the fluorescence intensity of 0.01–0.3 nM for linear fitting (Figure 6b) and calculated the detection limit to be 8.6 pM using the following formula, where $C_{L}$ is the lowest concentration, $X_{L}$ is minimum analysis signal, ${\overset{-}{X}}_{b}$ is blank average and m is the slope of the linear fitting curve.


$$C_{L}=\left(X_{L}-{\overset{-}{X}}_{b}\right)/m$$

The following Table 2 lists the detection limit and detection range of other detection methods proposed in recent years. The table illustrates that our method has a relatively large detection range and a low detection limit.

### 3.6. Selectivity of the miR-625-5p Assay

**Figure 7:**
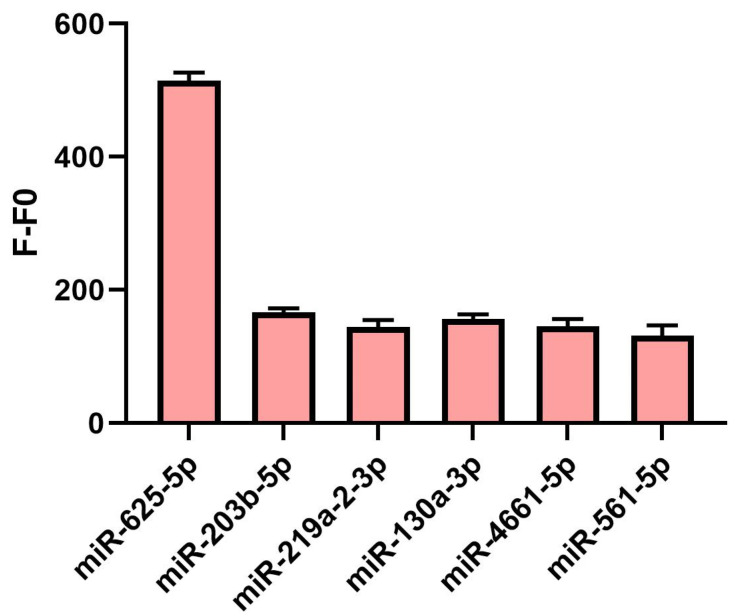
The specificity assay of miR-625-5p. The concentration of miR-625-5p and other interfering miRNAs were 100 nM. All data are presented as mean ± SD. Each assay was performed in triplicate.

In order to better illustrate the specificity of our detection method, we introduced five other miRNAs as an interference, and the fluorescence value of the detection minus that of the blank control considered as the relative fluorescence expression level of each experimental group. The result showed that the relative fluorescence intensity of the interference group was relatively similar and remained at a low level (Figure 7). However, the miR-625-5p group showed a significantly higher fluorescence intensity than the others. This confirms that our detection method has reached the expected specificity.

### 3.7. Assay of miR-625-5p in Biologic Samples

**Figure 8:**
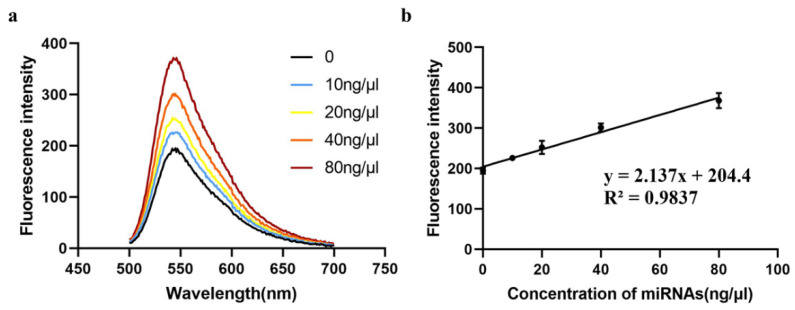
The assay of biological samples. (**a**) The fluorescence emission spectra of samples with different concentrations of miRNAs ranging from 0 to 80 ng/μL; (**b**) the plot of correlation between concentration of miRNAs and fluorescence intensity. The concentrations of miRNAs of HT-29 cells were 0, 10, 20, 40, 80 ng/μL. The fluorescence intensities of different concentrations are presented as mean ± SD. Each concentration was performed in triplicate.

In order to verify the practicability of this method, we selected HT-29 cells to extract their miRNAs, and diluted the sample concentration to 10, 20, 40 and 80 ng/μL. Additionally, no miRNA was added to the control. After the reaction was carried out according to our method, the fluorescence intensity of each sample concentration was detected. The result showed that the curves corresponding to different concentrations could be well-differentiated (Figure 8a) and the concentration ranging from 0 to 80 ng/μL had a linear relationship with the fluorescence intensity (Figure 8b), indicating that our method can be applied to detect real samples with different concentrations of miR-625-5p.

## 4. Discussion

In this study, we developed a novel method for miR-625-5p detection based on the basic principle of the isothermal amplification strand displacement reaction combined with SYBR Gold. Compared with the traditional RT-qPCR method, this method is simple, rapid and easy to operate. It does not need to rely on complex instruments and reaction conditions. Except for the process of hairpin formation and enzyme inactivation, which require a change in the temperature, the other reactions are basically completed at 37 °C. In order to further reduce the cost, we also optimized the amount of KF polymerase and Nt.BbvCI enzyme and the concentration of dNTPs. It is worth mentioning that compared with the existing strand replacement reaction, we did not use straight-strand cDNA in this study, but designed a hairpin sequence to further improve the specificity of the reaction and reduce nonspecific amplification reactions. The introduction of some interfering miRNAs also confirmed that the specificity of our method is high. In addition, for the subsequent strand replacement amplification reaction, we also changed the original one-step mixing reaction to a two-step reaction, so that the hairpin sequence would have sufficient time to open the hairpin structure and combine with the target sequence to enhance the reaction effect.

Through further experiments, we determined that the detection limit of this method is 8.6 pM, and the detection range is 0.01–200 nM. Compared with some existing methods, there is certain progress. Although there are some methods with a lower detection limit or a wider detection range, any method will have limitations. In the future, we can also combine some other methods to improve it, such as introducing special materials such as graphene to reduce the background.

Finally, in order to test the practicability of this method, we extracted miRNA from HT-29 cells and set different concentration gradients for detection using our method. The results showed that our method could well-distinguish biologic samples with different concentrations, showing a linear relationship in the concentration range of 0–80 ng/μL. According to this, we achieved the quantitative analysis of biologic samples by detecting the fluorescence intensity. This indicates that our method has broad clinical application prospects in the future, for example, we can apply this method to liquid biopsies for detecting miRNA biomarkers, which will contribute to the clinical diagnosis of colorectal cancer.

## 5. Conclusions

In summary, we identified miR-625-5p as a novel potential biomarker of CRC through bioinformatic analysis, clinical validation and phenotypic experiments, and developed a novel detection method based on the strand displacement amplification strategy for miR-625-5p. Compared with previous detection methods, our method has a simple operation and reaction conditions with great efficiency and sensitivity. The detection range is from 0.01 to 200 nM with a detection limit of 8.6 pM. Due to the application of a hairpin sequence, the method has high specificity. Additionally, in a real biological sample assay, it could well-distinguish between different concentrations. Our method will have broad application prospects in the clinical diagnosis of CRC biomarkers.

## Figures and Tables

**Table 1:** The required sequences of our detection method.

Sequence Name	Sequence (5′-3′)
Hairpin (HP)	CTATAGTCCAAACTATGCTGAGGGGACTATAGAACTTTCCCCCT
miR-625-5p	AGGGGGAAAGUUCUAUAGUCC
miR-625-5p (DNA)	AGGGGGAAAGTTCTATAGTCC

**Table 2:** Comparison of different detection methods.

Method	Materials	LOD (pM)	Detection Range (nM)	Reference
Electrochemical	MnO_2_ nanoflake	250	0.4–100	[31]
Fluorescence	2-Aminopurine/ThT	72	0.5–50	[32]
Fluorescence	Framework nucleic acid	40	0–500	[33]
Fluorescence	Ag nanocluster	60	0.1–8000	[34]
Fluorescence	DNA nanomachine	80	0.1–10	[35]
Fluorescence	SYBR Gold	8.6	0.01–200	This study

## Data Availability

Not applicable.
